# RE: Snake venom preparation for drug-resistant human immunodeficiency virus

**DOI:** 10.4103/0256-4947.51791

**Published:** 2009

**Authors:** Ramachandran Meenakshisundaram, Alagappan Uma, Ponniah Thirumalaikolundu Subramanian

**Affiliations:** a Institute of Internal Medicine, Madras Medical College, Chennai, India; b Institute of Microbiology, Madurai Medical College, Madurai, India; c Institute of Internal Medicine, The Tamil Nadu Dr. M.G. Ramachandran Medical University, Chennai, India

**To the Editor:**

Recently the usefulness of snake venom preparation in the treatment of drug-resistant human immunodeficiency virus was demonstrated in clinical practice for the first time by Alrajhi and Almohaizeie.^1^ This represents an emerging concept in the era of anti-retroviral therapy (ART). The authors have reported that snake venom might have enhanced the activity of ART or independently through some of its components. With reference to mechanisms of action, other components of snake venom such as the sequence homology between venom and HIV-1 gp 120^2^ and protease^3^ might also have contributed to the enhanced activity of ART at different levels.
